# A retrospective 11-year study on lip lesions attended at an oral diagnostic service

**DOI:** 10.4317/medoral.23390

**Published:** 2020-02-10

**Authors:** Caio César da Silva Barros, Cristianne Kalinne Santos Medeiros, Larissa Santos Amaral Rolim, Israel Leal Cavalcante, Pedro Paulo de Andrade Santos, Éricka Janine Dantas da Silveira, Patrícia Teixeira de Oliveira

**Affiliations:** 1 DDS, MSc, PhD Student. Postgraduate Program in Dental Sciences, Department of Dentistry, Federal University of Rio Grande do Norte, Natal, RN, Brazil; 2 DDS, MSc, Professor. Department of Dentistry, University of Fortaleza, Fortaleza, CE, Brazil; 3 DDS, MSc, PhD, Professor. Postgraduate Program in Dental Sciences, Morphology Department, Federal University of Rio Grande do Norte, Natal, RN, Brazil; 4 DDS, MSc, PhD, Professor. Postgraduate Program in Dental Sciences, Department of Dentistry, Federal University of Rio Grande do Norte, Natal, RN, Brazil

## Abstract

**Background:**

The objective of this study was to describe the upper and lower lip lesion occurrence in an oral diagnostic service.

**Material and Methods:**

Retrospective descriptive sectional study was performed. Clinical records were obtained from the archives of an Oral Diagnostic Service referral center between 2006 and 2016. Data such as gender, age, anatomical location, and diagnosis were collected and categorized. The collected data were submitted to a descriptive analysis and Pearson's chi-square test (*p* ≤ 0.05).

**Results:**

A total of 587 patient records of lip lesions were analyzed. Most lesions were diagnosed in female (52.1%) and adults (56.9%) patients in the lower lip (76.2%). Among all lip lesions, the reactive/inflammatory lesions (n = 238; 40.5%) and oral potentially malignant disorders (n = 164; 28%) were the most frequent group lesions. Mucocele (n = 147; 25%), actinic cheilitis (n = 136; 23.1%) and vascular lesions (n = 51; 8.7%) were the most frequent lesion in the sample. Actinic cheilitis was significant in relation to gender (*p* < 0.001), all three most frequent lesions were significant in concerning to age group and anatomical site.

**Conclusions:**

Mucocele was the most common lower lip lesion in all age groups, followed by actinic cheilitis and vascular lesions, which mainly affected adults and the elderly.

** Key words:**Lip, lip lesions, oral diseases, epidemiology.

## Introduction

Lips may be the site of clinical and pathological changes related to a wide spectrum of etiologies, ranging from traumatic, inflammatory and infectious lesions to malignant neoplasms (1-4). This anatomical site can host up to 25.7% of oral lesions, as well as a quarter of oral cancers, representing an important numerical data regarding diseases that affect oral tissues. The diagnosis of such a wide range of conditions should be cautious, taking into consideration their clinical aspects and, when appropriate, biopsies (5-12).

An important fact that should be taken into consideration when diagnosing lip lesions is the knowledge of their relative occurrence frequency (13). However, few studies on their prevalence in this anatomical site are available and, when performed, the emphasis is given to certain age groups and histopathological lesion diagnoses. In this respect, lesions that allow for diagnosis according to their clinical aspects and do not require biopsies are excluded.

Additionally, it is also necessary to obtain information about lip lesion distribution in relation to patient gender, age, anatomical location and risk factors, in order to determine the actual occurrence of such conditions. Broader studies taking into account lip lesion heterogeneity and including patients from all age groups are important to understand the actual occurrence of these lesions. In this context, the aim of the present study was to investigate the occurrence and clinicopathological features of diagnostic lip lesions in patients treated at a referral oral diagnostic service.

## Material and Methods

This is a retrospective sectional study investigating upper and lower lip lesion occurrence. Clinical records of patients treated at the Oral Diagnostic Service belonging to the Dentistry Department at the Federal University of Rio Grande do Norte (UFRN) (Brazil) from January 2006 to December 2016 were analyzed.

A medical record analysis was performed simultaneously by two researchers with experience in treating patients with mouth lesions. Adequately completed medical records of patients of all ages who presented an upper and/or lower lip lesion were included in the study. Medical records that did not meet these criteria were excluded from the sample. Data regarding gender, age, anatomic site of the lesion, diagnosis and recurrence were obtained. For biopsy cases, the histopathological analysis result was considered as the final diagnosis.

Diagnostic lesions were categorized according to their etiology into reactional/inflammatory, infectious, benign neoplasms, malignant neoplasms, and oral potentially malignant disorders (1,9). A patient age range categorization was created to facilitate the analysis, namely children (0-9 years), adolescents (10-19 years), adults (20-59 years) and elderly (over 60 years), according to World Health Organization criteria (14,15). Regarding the anatomic site, the following locations were considered: upper lip, lower lip and lip commissure.

The collected data were analyzed using the SPSS for Windows software (Statistical Package for Social Sciences; IBM, Chicago, IL, USA), version 22.0 and the results were submitted to a descriptive analysis and Pearson's chi-square test at a significance level of 5 % for the most frequent lesions.

## Results

Out of a total of 5,511 patient records, 587 presented lip lesions, representing an occurrence of 10.7%. The results regarding non-neoplastic lesions and neoplasms diagnoses, patient gender, and age group are described in Table 1 and Table 2, respectively.

Most lesions were diagnosed in female and adults. The most frequent lesions in the sample were mucocele (n = 147; 25%), actinic cheilitis (n = 136; 23.1%) and vascular lesions (n = 51; 8.7%). The lower lip was the most affected site (n = 447; 76.2%) (Table 3). Regarding recurrence, only 3.6% of the 587 analyzed cases presented lesion recurrence.

In the association test between gender, age group and anatomic site with the most frequent lesions (mucocele, actinic cheilitis, and vascular lesions), only actinic cheilitis displayed statistical significance concerning gender (*p* < 0.001). There was also statistically significant difference between mucocele, actinic cheilitis and vascular lesions with the age group (*p* < 0.001; *p* < 0.001; *p* < 0.001, respectively) and anatomic site (*p* < 0.001; *p* < 0.001; *p* = 0.002, respectively).

## Discussion

The lips represent an important lesion site when compared to other mouth sites, especially the lower lip, which was the main lesion site in this study, for 76.2% of all lesions. These data are similar to those reported by Osterne *et al*. (9), in which the authors analyzed 1,034 lip biopsy cases (16.6%) and observed that the lower lip is the most affected region (65.9%).

Reactional/inflammatory lesions were the most frequent, followed by potentially malignant disorders which together accounted for about two thirds of all lesions. Regarding the type of lesion, previous studies (9,16) also observed that mucocele is the most frequent, and the findings reported herein corroborate these data for all age groups (*p* < 0.001). We believe that this result is related to constant trauma in this region, especially in the lower lip, which was the most significantly affected herein (*p* < 0.001). Mucocele is an inflammatory lesion characterized by rupture or damage to the salivary gland duct, leading to mucus leakage into the surrounding tissues. Treatment is carried out by the surgical removal of the lesion and the affected gland, generally presenting an excellent prognosis. In this study, 57.1% of the assessed cases presented recurrence, probably due to the partial removal of the affected glands.

**Table 1:**
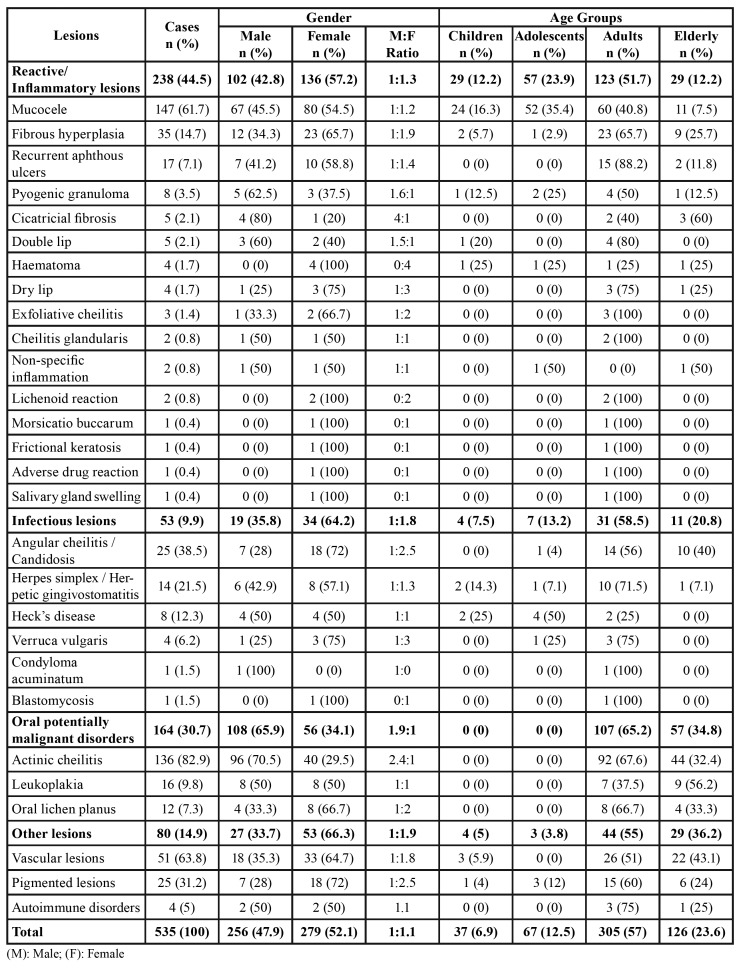
Gender and age group distribution of lip non-neoplastic lesions.

**Table 2:**
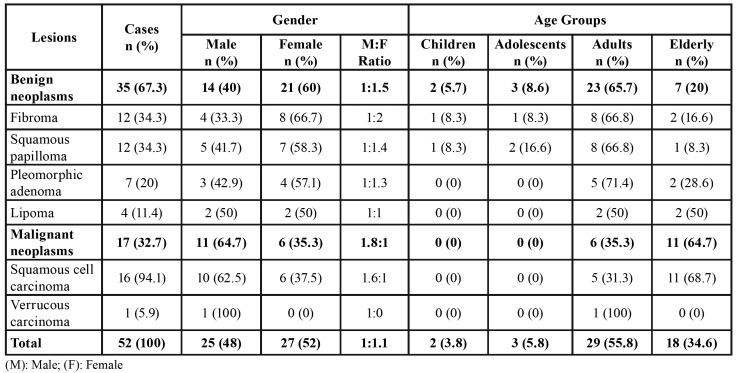
Gender and age group distribution of lip benign and malignant neoplasms.

**Table 3:**
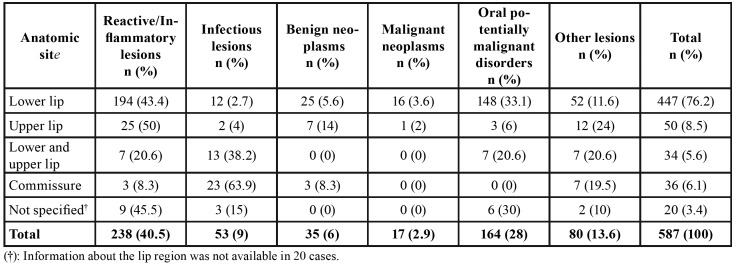
Distribution of lip lesions according to anatomic site.

The second most frequent lesion in the sample was actinic cheilitis, affecting almost exclusively the lower lip (*p* = 0.001) of adult and elderly patients (*p* < 0.001). This finding was expected, as actinic cheilitis is a chronic condition associated with prolonged exposure to solar radiation and develops insidiously over time. It is noteworthy that most individuals with this condition reported professional occupations related to sun exposure, justifying the high actinic cheilitis occurrence in this sample.

It is important to note that the presence of actinic cheilitis raises concerns regarding possible evolution to lower lip squamous cell carcinoma (LLSCC). In this study, LLSCC represented almost all of the malignant neoplasms, affecting mainly the lower lip of elderly men. No LLSCC were found on the upper lip, as it rarely affects this location (1,4,8-10,12). Probably, LLSCC does not affect the upper lip due to its anatomical position, less exposed to solar radiation than the lower lip.

Other oral potentially malignant disorders such as leukoplakia and oral lichen planus were also observed in this study, although the lower lip does not represent a frequent occurrence site for these lesions, as evidenced in other studies (2,11,13,17).

Vascular lesions are also frequently found on the lips, as reported by Ntomouchtsis *et al*. (13), who analyzed 140 benign lip lesion histopathological samples, reporting hemangioma as the most frequent lesion. In the present study, vascular lesions represented by vascular malformations and hemangioma corresponded to the third most frequent occurrence in the evaluated samples.

Benign neoplasms were less frequently observed in the present study, at 6.0% of the total analyzed cases. Fibroma and squamous papilloma were the two most common lesions in this category, affecting mainly adult individuals. Osterne *et al*. (9) indicated that hemangioma, followed by squamous papilloma, are the most common benign lip neoplasms, although in the present study hemangioma was not categorized, because the authors do not consider this lesion as a true neoplasm, but instead a developmental disorder.

The occurrence of lip lesions in patients treated at the Oral Diagnostic Service (UFRN) was assessed, analyzing both exclusively clinically diagnosed and biopsied lesions. The inclusion of eminently clinical lesions allows for data collection often not considered in most studies, which mainly analyze biopsied lesions (2,13). In this respect, infectious lesions representing 9% of the assessed sample could be evaluated. Angular cheilitis, a *Candida* injury that may or may not be associated with *Staphylococcus aureus*, was the most common infectious lesion, especially in adults and the elderly, followed by Herpes simplex virus infections, which corresponded to the second most frequent infectious lesion in this study. These lesions would not be considered if biopsies only were to be considered.

Our study was performed to investigate the occurrence of lip lesions on a referral oral diagnostic service in the Brazilian northeast. Clinical data seems the most reliable characterization of the occurrence of these lesions and, thus, permit dentists to become more familiarized with the diagnosis of these diseases at the time of initial diagnosis, which is a crucial time to establish the most likely differential diagnoses and, in this manner, being able to properly guide the pathologist who may eventually receive the biopsy. Occurrence studies are important to understand lower lip lesion frequency, although it is important to note that the data from the present study cannot be extrapolated to the general population, since medical records from a specific set of samples were analyzed. It should also be considered that adults and the elderly accounted for over half of the patients treated and that children and adolescents comprised a low sample representation in the present study. Therefore, lesion occurrence analyses in these groups should be carefully conducted.

## Conclusions

Mucocele was the most common lower lip lesion in all age groups, followed by actinic cheilitis, which mainly affected adults and the elderly. Understanding the occurrence, etiology and clinical characteristics of lesions affecting this anatomical site is paramount so that preventive and therapeutic measures can be promptly implemented.
